# A New Finite-Time Observer for Nonlinear Systems: Applications to Synchronization of Lorenz-Like Systems

**DOI:** 10.1155/2016/8342089

**Published:** 2016-09-22

**Authors:** Ricardo Aguilar-López, Juan L. Mata-Machuca

**Affiliations:** ^1^CINVESTAV-IPN, Departamento de Biotecnología y Bioingeniería, Av. IPN 2508, Col. San Pedro Zacatenco, Del. Gustavo A. Madero, Ciudad de México, Mexico; ^2^Instituto Politécnico Nacional, Unidad Profesional Interdisciplinaria en Ingeniería y Tecnologías Avanzadas, Academia de Mecatrónica, Av. IPN 2580, Col. Laguna Ticomán, Del. Gustavo A. Madero, Ciudad de México, Mexico

## Abstract

This paper proposes a synchronization methodology of two chaotic oscillators under the framework of identical synchronization and master-slave configuration. The proposed methodology is based on state observer design under the frame of control theory; the observer structure provides finite-time synchronization convergence by cancelling the upper bounds of the main nonlinearities of the chaotic oscillator. The above is showed via an analysis of the dynamic of the so called synchronization error. Numerical experiments corroborate the satisfactory results of the proposed scheme.

## 1. Introduction

Generally, nonlinear systems display complex dynamic behavior as steady state multiplicity, instabilities, complex oscillations, and so on, under different initial conditions, external disturbances, and time-varying parameters, leading to chaotic dynamic behaviors. However, besides the scientific interest on the study and analysis of nonlinear system with exotic dynamic behaviors, the applications for engineering purposes have been growing. Among these engineering applications, the employment of complex analysis for transport phenomena, chemical reacting systems, electronic industry, and synchronization technique for secure data transmission are actually very important [1–4].

In particular, the synchronization of chaotic oscillator is important for secure data transmission. Between several types of synchronization, one of the simplest and frequently studied types is the so called identical synchronization (IS). In this case the main purpose is to synchronize two or more chaotic oscillators with the same topology, which are coupled via an output injection of the measured signal from the master oscillator [5, 6]. The above has been analyzed with control theory techniques under the framework of nonlinear observers, where asymptotic, sliding-mode, finite-time, high gain observers have been applied for synchronization purposes [7–9].

In this work an identical synchronization technique for a master-slave configuration employing a class of nonlinear coupling of the measured signal to the slave system is proposed, in order to generate finite-time synchronization. The finite-time synchronization convergence is analyzed via the dynamic of the so called synchronization error under the assumptions that the upper bounds of the chaotic oscillators are known.

The rest of this work is organized as follows. In Section 2 the problem statement is described and the observer design is presented; the finite-time convergence is proved. In Section 3 the proposed methodology is applied in the synchronization of the hyperchaotic Lorenz-Stenflo system with success. Finally, in Section 4 the synchronization of the hyperchaotic Lorenz-Haken system is given.

## 2. Observer Design and Finite-Time Convergence

Let us consider the following general state space model:

(1) x˙=fx,x0=x0,y=hx,

where *x* = [*x*_1_, *x*_2_,…, *x*_*n*_]^*T*^ ∈ *Ω* ⊂ *ℝ* ^*n*^ is the states variable vector, *y* ∈ *Ω* ⊂ *ℝ* ^*n*^ is the corresponding measured output vector, *f* : *Ω* → *Ω* is a nonlinear differentiable vector function, and *f*(*x*) = [*f*_1_(*x*), *f*_2_(*x*),…, *f*_*n*_(*x*)]^*T*^, with initial conditions *x*(0) = *x*_0_ ∈ *Ω* ⊂ *ℝ* ^*n*^.

It is assumed that all trajectories of the state vector *x* of system (1) are bounded, considering the set *Ω* ⊂ *ℝ* ^*n*^ as the corresponding physical realizable domain, such that *Ω* = {*x*∣‖*x*‖ ≤ *x*_max_}. In most practical cases, *Ω* will be an open connected relatively compact subset of *ℝ* ^*n*^, and in the ideal cases, *Ω* will be invariant under the dynamics of system (1).

In the synchronization scheme, system (1) is considered as the master system.

Now let us propose a dynamical system to be synchronized with master system (1), which will be the slave system:

(2) $$\begin{array}{c} {\dot{\hat{x}}}_{i}=f_{i}\left(\;\hat{x}\;\right)-k_{1i}\left(\;{\left|\;\varepsilon_{i}\;\right|}^{-1/p}-k_{2i}\;\right) \end{array}$$

for *i* ∈ {1,2,…, *n*}, where ${\hat{x}}_{i}$ is the *i*th state variable of the slave system, $\varepsilon_{i}=x_{i}-{\hat{x}}_{i}$ is defined as the synchronization error, *p* > 1 and it is considered an odd integer, and *k*_1*i*_ and *k*_2*i*_ are positive constants.

Now we establish the analysis of the synchronization error and its finite-time convergence.

**Proposition 1 .**

Let master system (1), and consider slave system (2), where the following conditions are fulfilled:

(A1) There exists a constant *ℱ*_*i*_ ∈ *ℝ* ^+^ such that
  (3) $$\begin{array}{c} \left\|\;f_{i}\left(\;x\;\right)-f_{i}\left(\;\hat{x}\;\right)\;\right\|\leq F_{i}, \end{array}$$
 for all $\;x,\hat{x}\in \Omega \subset {\mathbb{R}}^{n}$.
(A2) The slave gains *k*_1*i*_ and *k*_2*i*_ are chosen such that
  (4) $$\begin{array}{c} F_{i}≅k_{1i}k_{2i}. \end{array}$$

Then, dynamic system (2) acts as a finite-time state observer for system (1), where the finite-time convergency is given by

(5) $$\begin{array}{c} t_{ft}=p\frac{{\left|\varepsilon_{0i}\right|}^{1/p}}{k_{1i}} \end{array}$$

with *ε*_*i*_(0) = *ε*_0*i*_.

**Proof**

The dynamic modeling of the estimation error dynamics is developed employing (1) and (2) as

(6) $$\begin{array}{c} {\dot{\varepsilon }}_{i}=f_{i}\left(\;x\;\right)-f_{i}\left(\;\hat{x}\;\right)+k_{1i}\left(\;{\left|\;\varepsilon_{i}\;\right|}^{-1/p}-k_{2i}\;\right). \end{array}$$

Applying the Cauchy-Schwartz inequality and (A1) to (6),

(7) $$\begin{array}{c} \left|\;{\dot{\varepsilon }}_{i}\;\right|\leq F_{i}-k_{1i}k_{2i}+k_{1i}{\left|\;\varepsilon_{i}\;\right|}^{-1/p}. \end{array}$$

Now, considering assumption (A2),

(8) $$\begin{array}{c} \left|\;{\dot{\varepsilon }}_{i}\;\right|\leq k_{1i}{\left|\;\varepsilon_{i}\;\right|}^{-1/p}. \end{array}$$

Notice that inequality (8) is a class of finite-time stabilization function, where the parameter *p* > 1 and it is considered an odd integer. Then the solution of inequality (8) is

(9) $$\begin{array}{c} \left|\;\varepsilon_{i}\;\right|\leq \mathrm{sign}\left(\;\varepsilon_{0i}\;\right){\left(\;\left|\;\varepsilon_{0i}\;\right|-k_{1i}\frac{t}{p}\;\right)}^{p}. \end{array}$$

At steady state (*ε*_*i*_(*t*) = 0),

(10) $$\begin{array}{c} t\geq p\frac{{\left|\varepsilon_{0i}\right|}^{1/p}}{k_{1i}}. \end{array}$$

Then, the finite-time convergency is given by

(11) $$\begin{array}{c} t_{ft}=p\frac{{\left|\varepsilon_{0i}\right|}^{1/p}}{k_{1i}}. \end{array}$$

## 3. Synchronization of the Hyperchaotic Lorenz-Stenflo System

The Lorenz-Stenflo system is described as [10]

**Figure 1:**
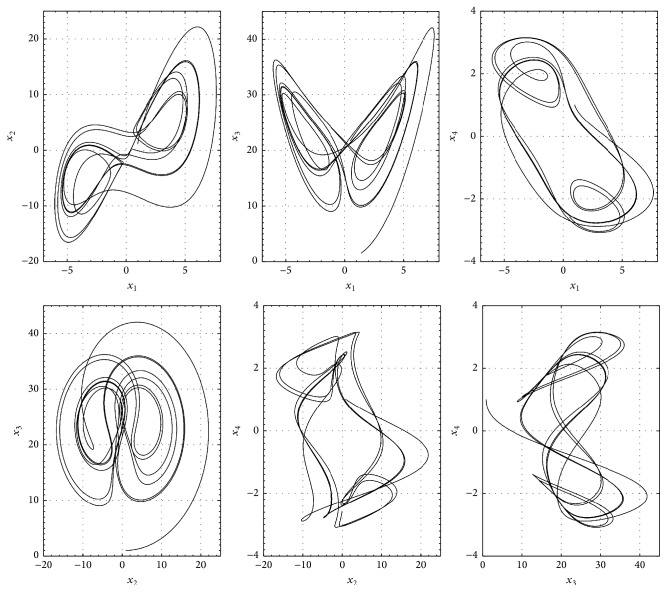
Phase space of the hyperchaotic Lorenz-Stenflo system (12).

(12) x˙1=ax2−ax1+dx4,x˙2=cx1−x1x3−x2,x˙3=x1x2−bx3,x˙4=−x1−ax4,

where *a*, *b*, *c*, and *d* are positive parameters. With *a* = 1, *b* = 0.7, *c* = 26, and *d* = 1.5 system (12) exhibits hyperchaotic behavior, as is shown in Figure 1. For the numerical results we have taken the initial conditions *x*_1_(0) = 1, *x*_2_(0) = 1, *x*_3_(0) = 1, and *x*_4_(0) = 1. In this section, system (12) is viewed as the master system. We can see from Figure 1 that the whole state solution of system (12) is bounded; therefore, we can claim that assumption (A1) is completely fulfilled.

As slave system, we consider the dynamical system given by

(13) x^˙1=ax^2−ax^1+dx^4−k11ε1−1/p−k21,x^˙2=cx^1−x^1x^3−x^2−k12ε2−1/p−k22,x^˙3=x^1x^2−bx^3−k13ε3−1/p−k23,x^˙4=−x^1−ax^4−k14ε4−1/p−k24

with *a* = 1, *b* = 0.7, *c* = 26, *d* = 1.5, and *p* = 3.

The numerical bounds for the trajectories of the Lorenz-Stenflo system (12) have been estimated in [10]. It was proved that system (12) has ultimate bounds and its trajectories belong to an invariant set.

For the tuning of the slave gains of system (13) we include Table 1 in order to find the upper bounds *ℱ*_1_, *ℱ*_2_, *ℱ*_3_, and *ℱ*_4_, corresponding to assumption (A1).

According to the numerical results of Table 1, the values of *ℱ* are approximated as

(14) F1≅20,F2≅70,F3≅100,F4≅5.

Then, applying assumption (A2), the slave gains are fixed as

(15) k11=20,k12=70,k13=100,k14=5,k21=1,k22=1,k23=1,k24=1.

**Figure 2:**
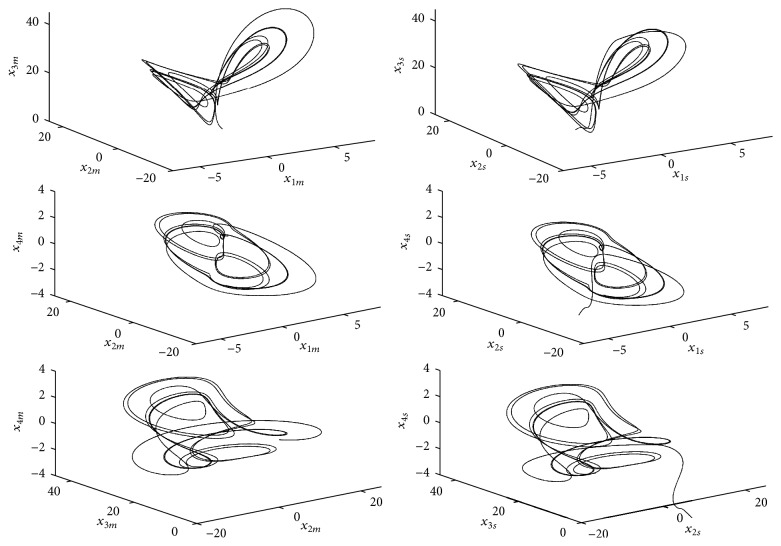
Phase space of the synchronization of the hyperchaotic Lorenz-Stenflo system (12) and its observer (13). The subscripts *m* and *s* represent the variables of master and slave systems (12) and (13), respectively.

**Figure 3:**
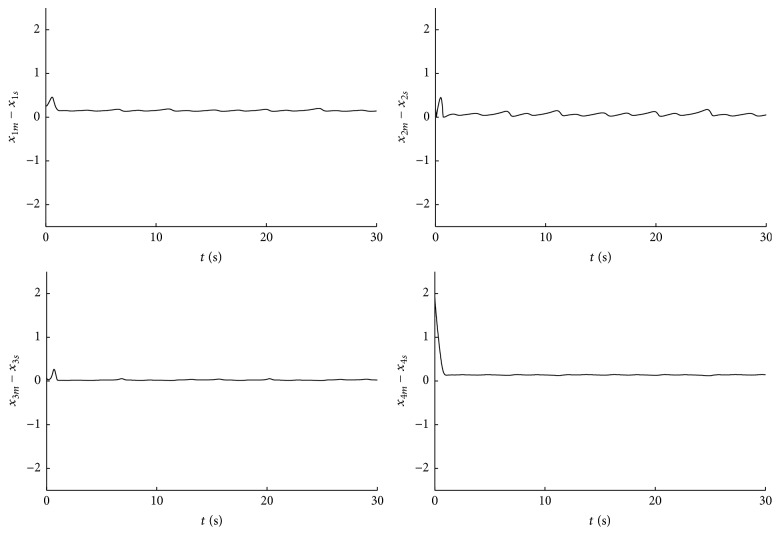
Synchronization errors.

Some numerical simulations are performed using the set-up of parameters as *a* = 1, *b* = 0.7, *c* = 26, and *d* = 1.5 and fixing the slave system exponent as *p* = 3. We consider the following initial conditions to the master system *x*_1_(0) = 1, *x*_2_(0) = 1, *x*_3_(0) = 1, and *x*_4_(0) = 1 and the initial conditions to the slave system ${\hat{x}}_{1}(0)=-1$, ${\hat{x}}_{2}(0)=5$, ${\hat{x}}_{3}(0)=-2$, and ${\hat{x}}_{4}(0)=-5$. The synchronization between master system (12) and slave system (13) is shown in Figure 2, where the convergence of the state estimates to the real states is depicted. The subscripts *m* and *s* represent the variables of master and slave systems (12) and (13), respectively. As we can note in Figure 3, the synchronization results achieved with the finite-time observer are good, where each image represents the corresponding synchronization error defined as

(16) ε1=x1m−x1s,ε2=x2m−x2s,ε3=x3m−x3s,ε4=x4m−x4s.

## 4. Synchronization of the Hyperchaotic Lorenz-Haken System

We illustrate the proposed synchronization scheme with another hyperchaotic system so called Lorenz-Haken, given by the following equations [11]:

(17) x˙1=ax2−ax1,x˙2=αx1−x2−cx3+ax32x1−x1x4,x˙3=βx1+cx2−x3−ax2x3x1,x˙4=−bx4+x1x2.

**Figure 4:**
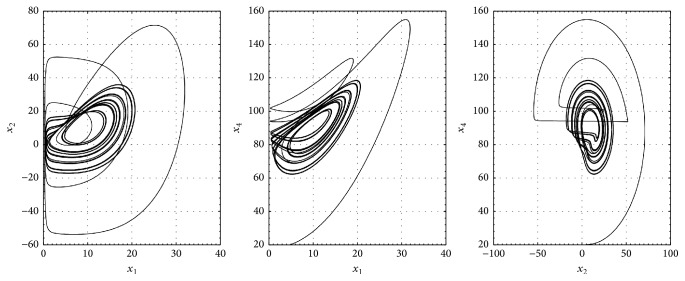
Phase space of the hyperchaotic Lorenz-Haken system (17).

Figure 4 shows the hyperchaotic behavior of system (17) for *a* = 6, *b* = 1.2, *c* = 2.5, *α* = 91, and *β* = −1.5, with initial conditions *x*_1_(0) = 5, *x*_2_(0) = 5, *x*_3_(0) = 4, and *x*_4_(0) = 20.

Consider Lorenz-Haken system (17), referred to as the master system, and let us propose the following slave system:

(18) x^˙1=ax^2−ax^1−k11ε1−1/p−k21,x^˙2=αx^1−x^2−cx^3+ax^32x^1−x^1x^4−k12ε1−1/p−k22,x^˙3=βx^1+cx^2−x^3−ax^2x^3x^1−k13ε1−1/p−k23,x^˙4=−bx^4+x^1x^2−k14ε1−1/p−k24

with *a* = 6, *b* = 1.2, *c* = 2.5, *α* = 91, *β* = −1.5, and *p* = 5.

Computer simulations have been carried out in order to test the effectiveness of the proposed synchronization strategy using the same set-up as above and fixing the slave system gains as

**Figure 5:**
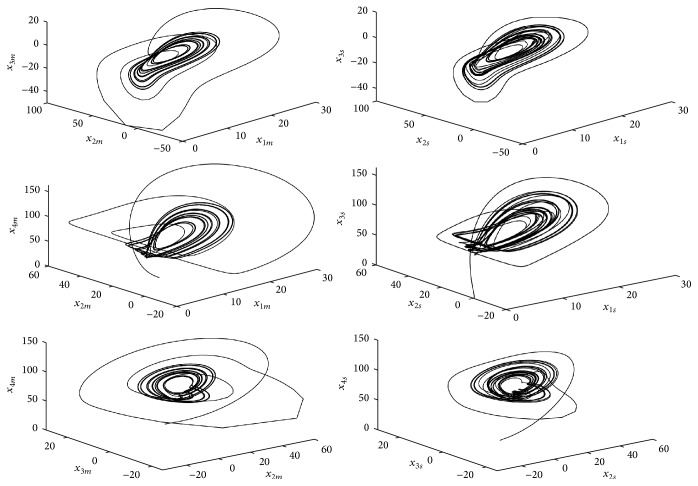
Phase portrait of the synchronization of the hyperchaotic Lorenz-Haken system (17) and its observer (18). The subscripts *m* and *s* represent the variables of master and slave systems (17) and (18), respectively.

(19) k11=50,k12=40,k13=50,k14=20,k21=1,k22=1,k23=1,k24=1

with the slave system initialized at ${\hat{x}}_{1}(0)=0.1$, ${\hat{x}}_{2}(0)=0$, ${\hat{x}}_{3}(0)=0$, and ${\hat{x}}_{4}(0)=0$. In Figure 5 we can see that the synchronization in phase portraits, that is, the trajectories of slave system (18), follows the trajectories of system (17).

## Figures and Tables

**Table 1:** Estimation of the upper bounds of assumption (A1).

Bounds of *x*	Bounds of $\hat{x}$	$\max\left(\left|f(x)-f(\hat{x})\right|\right)$
−6.0099 ≤ *x*_1_ ≤ 7.6721	$-7.1676\leq {\hat{x}}_{1}\leq 5.5370$	$\left|f_{1}\left(x\right)-f_{1}\left(\hat{x}\right)\right|\leq 20.2674$
−16.6322 ≤ *x*_2_ ≤ 22.0500	$-18.4823\leq {\hat{x}}_{2}\leq 18.8447$	$\left|f_{2}\left(x\right)-f_{2}\left(\hat{x}\right)\right|\leq 71.2132$
−1.0000 ≤ *x*_3_ ≤ 41.9295	$-2.0000\leq {\hat{x}}_{3}\leq 36.99$	$\left|f_{3}\left(x\right)-f_{3}\left(\hat{x}\right)\right|\leq 97.4952$
−3.0921 ≤ *x*_4_ ≤ 3.1359	$-5.0000\leq {\hat{x}}_{4}\leq 2.6915$	$\left|f_{4}\left(x\right)-f_{4}\left(\hat{x}\right)\right|\leq 4.9927$
